# The Louisiana State Board of Health

**Published:** 1898-01

**Authors:** 


### Abstracts and Selections.

### THE LOUISIANA STATE BOARD OF HEALTH.

### Resignation Forced by "Commercial Interests" so-called. A Cruel Sacrifice to Prejudice.

The JOURNAL believing that a cruel injustice has been done the State appointees on the Louisiana State Board of Health, reproduces here from the *Picayune* the letter of resignation of the five State appointees, together with their account of the origin, rise, progress and results of the recent epidemic in New Orleans. Some people do not know when they are well off. The Louisiana people will never get another board as capable, as unselfish, as earnest and zealous in the cause of public health—nor I may add—as successful in preventing an extensive epidemic as the one just gone to pieces in response to a popular clamor of soreheads. Let these growlers contrast the recent outbreak and its results to that of 1878, when there were 25,000 cases and 4000 deaths; this year there were less than 2000 cases and 300 deaths. If commerce suffered this year, what would it have suffered had this board been less vigilant and efficient, and let the epidemic attain the magnitude of that of 1878? It looks like child's play, petulence. The merchants smarting under losses (which, but for this board's activity, might have been trebled), pouted, and somebody had to suffer. Unlike the case of Abraham—who was preparing to sacrifice his son, when most opportunely there came galloping up a he-goat and offered his services as a substitute—there was no 'scapegoat in this instance, and these gentlemen had to be slaughtered.

The outcome of this affair—especially the proposition which these gentlemen say was made by or on behalf of commercial interests, to keep a double set of books; one true, one false, to deceive the press and the people—only emphasizes the charges that were made against certain New Orleans firms, that they were trying to smuggle goods (and with them infection) out of New Orleans into Texas and elsewhere, in violation of the law and with a reckless disregard of consequences that is criminal. Let the board be heard in its defense. The names of the prime movers in slaughtering these officers should be known and marked with a black cross mark:

The members of the board wished to give a clear and concise account of their actions in order that they be rightly understood by the public. In order to do this it was thought best to include the original letter mailed to Governor Foster, giving the reasons for their resignation.

The members of the Board of Health feel that they have conscientiously done their duty in their place of public trust, and that their actions have been wrongly interpreted by the public. They take this occasion of stating fully their side of the important question.

The letter to Governor Foster was as follows:

New Orleans, La., Dec. 6, 1897.

*To His Excellency, Murphy J. Foster, Governor of Louisiana:*

Sir:—We have the honor herewith to resign our commissions as members of the Board of Health of the State of Louisiana.

In so doing, we wish it to be understood that we are not moved by the clamor of a frenzied public, but by a respectful wish to spare your excellency the possible embarrassment of being importuned by certain commercial exchanges to ask for the resignations of sworn officers holding your commissions, because the members of those exchanges, smarting under recent business losses, and stimulated by hostile newspaper criticisms of this board, have assumed the prerogative of voicing public sentiment.

Our official conduct, throughout, will bear the closest investigation, and we feel that we can rely on truthful history for that vindication before the people, which, in the present disturbed condition of the public mind, it would be vain to attempt.

It is necessary, however, for the immediate official vindication of the retiring members that certain facts, bearing directly on the action of the board, should be set forth here.

The accompanying statement gives these facts in detail, and in justice to us, we trust that you will patiently read the same. By examining the dates referred to, it will be seen that the utmost vigilance and promptness marked every step taken by this board.

Finally, with the consciousness of having labored faithfully, as a board, all these years to discharge the responsibilities we had assumed to our city and State, and to the entire Mississippi valley, we now retire from that service, with the hope that our immediate successors may be more fortunate.

Respectfully, your obedient servants,

S. R. Olliphant, M. D.,
Geo. K. Pratt, M. D.,
Edw. S. Maunsell,
M. V. Richard, M. D.,
T. W. Dyer.

Statement annexed and made part of the resignations of members of the Board of Health.

Reviewing the fever from the first suspicion, we give below the published statement of the President, which covers the history of the board's action up to the 6th of September:

The first intimation of yellow fever in this country to reach this office came through a telegram of inquiry, dated August 16, referring the President of the Louisiana Board of Health to a letter from a doctor at that time unknown to me, which letter had not been received. I replied at once, asserting that there was no yellow fever in New Orleans, and asking who the doctor was. He answered, on August 17, that he was one of their prominent physicians, and again referred me to his letter. On August 18, in the afternoon, I received the letter spoken of, which informed me of a patient under treatment, represented at that time as having left New Orleans on the 12th, and who had received medical treatment en route. He had been previously under the treatment of a physician in New Orleans, whose name is given in the letter (Dr. E. S. Lewis.)

The case was pronounced one of undoubted yellow fever by the doctor in Louisville. The letter states that, owing to the incoherency of the patient, no definite history of the case could be obtained, and, further, states in a potscript that the patient had been in Ocean Springs one week before coming to Louisville.

Immediately on the receipt of this letter, on August 18, the office where this patient was employed was interviewed, and the information that he had been in Ocean Springs was verified. The New Orleans physician, referred to as having previously attended him, was sought, but found to be out of the city. It transpired later, however, that he had been under the treatment of another physician in this city for malarial toxæmia (Dr. John B. Hart). I have the written report of the latter physician attesting this fact. These facts were embodied in a letter to the physician in Louisville, dated August 19. In the meantime I received a telegram announcing the death of the patient in question, and at once wired a request that an autopsy be held. I received, on the 20th, a letter dated on the 18th, stating that, owing to the confusion attending the death, the body had been injected with embalming fluid before an autopsy could be obtained. The same letter inclosed clinical records of the case. In addition to the investigations made in the City of New Orleans, inquiry was made through a physician connected with the office (Dr. W. H. Woods, Chief Sanitary Inspector), who had just returned from a two weeks' vacation at Biloxi, as to the prevalence of any suspicious disease in that country. He assured me that he had met the physicians of both Ocean Springs and Biloxi frequently, and if there had been any fever of a suspicious nature he would most probably have heard of it.

On the 21st I received another letter, in the afternoon of that day, from Dr. Holloway, in Louisville. It was dated on the 19th. This letter conveyed information elicited from the wife of the deceased to the effect that the disease was contracted in a house at Ocean Springs, occupied, or visited, by Cuban refugees. On this information I determined at once to make a personal investigation, and proceeded on the early morning train of the next day, August 22 (Sunday), to Ocean Springs accompanied by a member of the board. Through the courtesy of the local profession and a prominent New Orleans physician (Dr. J. H. Bemiss) who was summering there, I was enabled to visit a number of cases of fever. In addition to the cases I saw, I learned that there had been in the practice of two physicians within the past six weeks as many as 400 cases of fever, without a single resultant death. This disease was considered as dengue, and that of a mild type, up to within a day of my visit. Some of the cases I saw were sufficiently ill to attract more than a passing notice. In view of the proximity of Ocean Springs to New Orleans, and the constant daily travel between the two places, I confess to having felt great surprise at the existence of a fever so extensively prevalent, and yet not even rumored in New Orleans.

Returning to New Orleans on the evening train, I at once took steps to get members of the board of experts to accompany me the next day to Ocean Springs, with a view to making a thorough clinical investigation. By 11 o'clock the next day, in company with three prominent physicians, Drs. O. Czarnowski, L. F. Salomon and G. F. Patton, experienced in yellow fever, I left New Orleans on the fast mail, specially delayed for our accommodation. I telegraphed Dr. Haralson, resident of Biloxi, a member of the State Board of Health of Mississippi, and asked him to join us, which he did. Arriving in Ocean Springs, we proceeded at once to investigate, and after seeing all the cases I had visited the day previous, and some others, these gentlemen submitted to me the following report:

"OCEAN SPRINGS, MISS., Aug. 23, 1897.

"*Dr. S. R. Olliphant, President of the Board of Health of Louisiana:*

"DEAR SIR:—The undersigned having been requested to investigate an epidemic of fever prevailing at this place, would report as follows:

"In company with Drs. J. H. Bemiss and O. L. Bailey, attending physicians, we visited and carefully examined eleven cases of the prevailing disease, of which we have been informed there have been during the past six or seven weeks over 400 cases, none fatal, except two or three complicated with preexisting organic trouble. After a careful inspection and examination of the aforesaid cases, which correspond in clinical history with other existing cases, and cases which have previously occurred, we are positive in our opinion that the disease is dengue, and that in no case, either in those seen by us or in cases whose history has been obtained from the attending physician, is there or has there been, any symptoms which would lead to even a suspicion of more serious disease. Signed by the entire medical commission present.

"LUCIEN F. SALOMON, M. D.,
"O. CZARNOWSKI, M. D.,
"Of the Board of Experts.
"G. F. PATTON, M. D.,
"Secretary of the Board of Health.
"A. H. HARALSON, M. D.,
"Member of the Mississippi State Board of Health.

"We, the undersigned, concur in the above diagnosis of the disease prevailing at Ocean Springs.

"O. L. BAILEY, M. D.,
"J. H. BEMISS, M. D."

I telegraphed to convene the board in special session awaiting our return. Arriving in New Orleans the report was submitted to the board. There was a great feeling of relief. The report was published in all the papers, and for a time being all occasion for apprehension appeared to have been allayed.

Further rumor relative to Ocean Springs reached this office. I telegraphed on the 27th, requesting the representative of the Mississippi State Board of Health to again investigate Ocean Springs, especially in regard to one patient whose name was connected with the rumor. He at once made the investigation and sent me the following dispatch in reply:

"OCEAN SPRINGS, MISS., Aug. 27, 1897.

"*Dr. S. R. Olliphant, President of the Louisiana State Board of Health:*

"In reply to your request, we have again investigated the fever at Ocean Springs, which is abating and absolutely without fatality. The conclusion arrived at was that it is not yellow fever. The case of Miss—— is free from suspicion. By invitation, Dr. Sanders, health officer of Alabama, joined us in the investigation, and shares the opinion with us.

"H. H. HARALSON, M. D.,
"Member of the Mississippi State Board of Health.
"O. L. BAILEY, M. D.,
"Attending Physician.
"W. H. SANDERS, M. D.,
"Health Officer of Alabama.
"J. H. BEMISS, M. D."

This is the first evidence I had of an investigation being prosecuted by Alabama authorities.

On September 1, 2 o'clock in the morning, I was rung up by telephone by a prominent physician of this city, Dr. E. T. Shepard, who stated that he had a suspicious case of fever on Soniat Street, and invited me to investigate the same with members of the board of experts, to meet at his house at 9 o'clock of the same morning. As this case had come from Ocean Springs, I purposely got together the same medical gentlemen who had visited Ocean Springs with me. We met promptly at 9 o'clock, at the doctor's house, and proceeded to the patient's residence. After a thorough examination and discussion of the case, the conclusion was reached that it was not yellow fever. On September 2, in the afternoon, the attending physician, Dr. E. T. Shepard, called at the Board of Health office, stating that he was not satisfied with the conditions of the patient, and that he invited further investigation on the part of the board of experts. At the same time he said he would have with him some medical friends of his own selection. The same gentlemen of the board of experts, with one other member, Dr. J. C. Bickham, added, met the attending physician, Dr. E. T. Shepard, that evening, who had with him two prominent practitioners of this city, Drs. F. W. Parham and F. Loeber, whom he had requested to participate in the consultation. There was a special meeting of the Board of Health that evening, convened to await the result of this conference, with a view to taking such action as might be considered necessary. I was authorized to state, on my arrival at the board's meeting, that this was not yellow fever. However, a part of this investigating commission would see the case again the next day, and give me a positive decision as to even suspicion. This further investigation would take into consideration the bacteriological examination of the blood, which would be made pending the time of their meeting the next morning. At 11 o'clock that night, the acting bacteriologist, Dr. J. J. Archinard, went from the office of the Board of Health and obtained from the patient a sample of his blood and completed his examination and submitted his report for the consultation of the next morning. After this consultation, one of the gentlemen, Dr. L. F. Salomon, reported to me that he was authorized to state, on behalf of his colleagues, that the case was absolutely not suspicious of yellow fever, and the report of the bacteriologist sustained the decision by reason of finding the characteristic organisms of malaria present in large quantities in the blood. In the face of this evidence, the Board of Health could not at that time consider this a case of yellow fever, and the house was never quarantined.

At a special meeting of the board, held Friday, September 3, to receive the report of the medical commission having in hand the matter of the Soniat Street case, it was determined that another investigation should be made at Ocean Springs, with a view of ascertaining the character of the prevailing disease and determining, if it was not yellow fever, what kind of fever it was, and what the cause of it. For this thorough investigation I took with me a physician experienced in yellow fever, Dr. S. G. Gill; the acting bacteriologist, J. J. Archinard; the chemist of the board, Dr. A. L. Metz; the chief sanitary inspector, Dr. W. H. Woods, and his assistant, Mr. T. C. Will, with a full equipment of instruments and material. We left Saturday, September 4, arriving at Ocean Springs in the evening. We found Dr. Sanders, the health officer of Alabama, and a representative of the United States Marine Hospital, Dr. Wasdin, already on the field, they having arrived by an earlier train that day from Mobile. They having had an opportunity of an autopsy during the day, were convinced that it was yellow fever, but, in deference to us they agreed to withhold their report or decision until another autopsy, which would evidently present itself within a few hours, should have offered us a similar opportunity of satisfying ourselves. The patient died Sunday night, and early Monday morning the expected autopsy was performed by the bacteriologist of this board in the presence of the medical gentlemen assembled. Unmistakable evidences of yellow fever having been revealed, we arrived at a unanimous verdict, and about 10 o'clock of the same morning I telegraphed this information to the Board of Health, and requested the board to be in session at 3 o'clock, the hour of my return. While yet at Ocean Springs, I received a telegram from a physician of this city, Dr. S. L. Theard, announcing the death of a patient from yellow fever on Rampart Street. These occurrences took place Monday, September 6.

The character of the Rampart street case was confirmed by an autopsy, and without waiting for the return of the president, the board was convened at 11 o'clock a. m. in special session. Acting on advice received from the president at Ocean Springs, a resolution was adopted declaring quarantine against all points on the Louisville & Nashville railroad west of Mobile, the same to go into effect immediately, with the proviso that the president be authorized to relieve such places as might be subsequently found to be free from infection. A copy of this resolution was sent by a special messenger to Mr. Charles Marshall, superintendent of the Louisville & Nashville railroad, and reached him about 1 o'clock Monday, September 6. Simultaneously notices were sent to the express company, and the baggage department, forbidding the bringing of express matter or baggage to this city. At 5:30 o'clock, the president having arrived, the board was again convened in special session to receive his report, and to consider what further measures should be adopted. The superintendent of the Louisville & Nashville railroad came before the board, and stated that the incoming trains had not stopped at Ocean Springs, and had been boarded at Biloxi and points this side by crowds of people, principally women and children, without any baggage whatever, and in defiance to opposition. These people were then on their way to New Orleans. In the discussion which ensued, it was argued that these people were New Orleans people, coming from points at that time not known to be infected, and that they might safely be admitted. The president, who had arrived on a special train, reported that Biloxi had already instituted quarantine against Ocean Springs, and that from conversation that day with representatives of the State Board of Health of Mississippi, resident in Biloxi, who had returned to Biloxi on the special train, he was informed that there was no suspicious case in Biloxi. The board, taking into consideration that there was nobody on these trains from a known infected point, felt justified in allowing these two train loads of people to return.

As this case has been a matter of such widespread comment, I have taken the trouble to reinvestigate the whole matter, and have been assured that absolutely no one from Ocean Springs came in on either of these two trains.

In answer to criticisms of the action of the board based upon the statement, the following additional explanations were submitted:

First—No train load of people was ever admitted to New Orleans from Ocean Springs after the diagnosis of the disease.

Second—At the time of the autopsy made by Drs. Sanders, Wasdin, Harralson and Bailey in the Tillman case, there was a division of opinion between them as to the nature of the disease. Two were of the opinion that it was yellow fever, and two were of the contrary opinion. Neither our experts nor any one of our board had seen the case or had been present at the autopsy, we being on the way to Ocean Springs at the time. Prior to, and at the time we were investigating, we learned positively that cases had been taken from Ocean Springs to New Orleans and Mobile, so that we were practically diagnosing the fever for New Orleans and Mobile. It will, therefore, be understood that we could not pronounce the disease yellow fever from an autopsy we never held, but which was held by gentlemen who disagreed among themselves, two holding one opinion and two another, and in the face of an opinion of some of our most distinguished physicians, who were recognized experts, that the prevailing disease was dengue. I submit, that to have accepted the diagnosis of a divided opinion in the Tillman case, and to have announced to the world that yellow fever was in Ocean Springs, and by implication in New Orleans and Mobile, which would have been the inevitable result based upon the opinion of these two physicians, would have precipitated a calamity which it was earnestly hoped we would be able to avert.

In conclusion, I submit that to have blindly accepted the diagnosis of yellow fever, based on the decision of two gentlemen present at the autopsy, disregarding the opinion of the other two, and absolutely discrediting the opinion of our experts, would have been in the highest degree irrational and culpable.

If the disease had been subsequently proven not to be yellow fever, the infliction of a needless panic, caused by such action, would have entailed upon all parties concerned the just condemnation of the entire country.

(I have since learned that Dr. Bailey was not present at the autopsy).

At the sessions of the Board of Health, which were held daily, advice and conference were offered by prominent and public-spirited citizens, by representatives of the exchanges, by the mayor and city council, and, later, by a committee from the Orleans Parish Medical Society.

A board of experts, composed of ten physicians of prominence and experience, and recognized by the profession and public as men of ability, were requested to co-operate with us from the beginning. It will be seen there was no lack of consultation.

The press also shared in our consultations, and for the time all seemed harmonious and absorbed in the great and united effort of averting a threatened calamity, such as befell us in 1878.

That these united efforts were not without effect we call attention to the fact, by comparison, that in 1878 there were 25,000 cases and over 4000 deaths; whereas in 1897 the grand total of cases reported did not reach 2000, nor the deaths 300.

While it may appear that the disease made its advent in the first days of September, it is in evidence that there were cases here early in August, and, it is claimed, as early as the latter part of June.

The recorded history of the disease shows that it does not burst forth all of a sudden, but it gathers volume and force after smoldering for a time. The events connected with the development of the disease at Edwards, Miss., present a remarkable verification of this fact. There the fever was introduced by a family from Ocean Springs as early as the 8th of August, and it was not until the middle of September that the disease attained any considerable headway. Such has been the invariable clinical history of the disease in the city of New Orleans in past epidemics.

The Board of Health has been criticised and severely censured for general inactivity and incompetency, for letting the fever into New Orleans, for reporting the cases that occurred, for not stamping out at once the disease and preventing its spread, and lastly, for telling the truth.

The last charge is the only one to which we plead guilty.

To take up the other charges seriatim:

The first has been fully covered by the statement of the President above quoted.

As regards the second charge, the board cannot reasonably be blamed for not preventing the entrance of the fever, which had already found its way unrecognized into the city, and was of such a mild type as not to attract the attention of the physicians of New Orleans. No cases came under the observation of the officials of the Board of Health, and no breath of suspicion was whispered concerning it by any of the profession. The Board of Health necessarily depends for its information as to the existence of infectious diseases upon the reports of practicing physicians.

As to the third charge, that of reporting cases, it is only necessary to reflect for a moment, and it will be realized that the practicing physicians of the city diagnosed and reported as in duty bound, the cases to the Board of Health in conformity with existing laws. Only in doubtful cases, so reported by attending physicians, were experts of the board sent to pass upon the true nature of the disease. It is but fair to state that these experts, selected from the foremost ranks of the medical profession, were in no manner in the employ of the board, but rendered their services gratis. Later, two experts, Drs. Wolf and Mioton, were employed, as the calls for this service became so numerous. The Board of Health only received the reports of and recorded the cases pronounced yellow fever by the medical practitioners of New Orleans. The medical officers of the board were absolutely prevented from practicing by reason of their onerous duties, and, therefore, did not undertake to treat cases of yellow fever. Our office simply received reports, and we had neither authority nor inclination to suppress or falsify our records.

The press and public are privileged by law to examine board of health records. When cases became alarmingly numerous, it was suggested that a double set of books might be kept. Such suggestions were repelled with the scorn they merited. Money may be the god and ledgers the prayer-books of some people, but if this State should ever have a Board of Health so weak as to be influenced by such sordid interests, we may expect quarantine against this city every summer.

Our board of experts is composed of the following-named gentlemen, all of whom are honest, honorable and competent physicians:

Drs. O. Czarnowski, John B. Elliott, A. Petit, L. F. Solomon, T. S. Kennedy, C. J. Bickham, Isidore Dyer, Y. R. McMonier, J. Toutre, and G. Devron. Later, through the courtesy and tender of the Orleans Parish Medical Society, their committee, composed of Drs. R. Matas, John Callan, F. W. Parham, A. Petit, and E. M. Dupavuier, were added to the list, making a board composed of fourteen prominent practitioners of medicine in this city.

In the numerous conferences that took place, it goes without saying that every individval idea could not be carried out. These gentlemen now admit, however, that respectful consideration was given to all suggestions. The board, feeling its own responsibility, as an official body, after full discussion on all points on which there was any difference of opinion, acted consistently, conscientiously and in line with what it deemed for the best interest of the city.

There was a difference of medical opinion as to the propriety of maintaining house quarantine as long as we did, but there were no differences about inaugurating this practice.

There was likewise a difference of opinion in the mind of the public and the press on this same subject; the two morning papers wrote editorials, strongly urging keeping up the fight on the lines originally mapped out. Some of the evening papers were opposed to this course; prominent business men and merchants, and the railroad men without exception, demanded no relaxation, while others declared it was a hardship and a useless expenditure of money. There was a time when the board seriously contemplated removing quarantine, by reason of lack of funds. At this juncture public-spirited citizens got together and appealed to the Governor and legislature for aid, and, as a result, $50,000 was placed at the disposal of the board. Could we, with any show of reason, under the circumstances, have given up the fight? To have done so would have been cowardly and a just cause of reproach. We would have declared to the world that the fever was epidemic and beyond our control, and would have entailed a calamity on this city far more extensive than our present misfortune will prove. That our efforts in restricting the disease were not more successful, is due, in a great measure, to lack of co-operation on the part of many of the physicians who were opposed to our sanitary methods and failed to report their cases. Some physicians who honorably reported their cases, stated that others were concealing cases for fear of offending their clients and losing their practice. These wilfully violated the law. This course, on the part of some physicians, tended, to a great extent, to foment opposition to the measures of the board. One family would naturally rebel against restrictions, when a neighbor, having the same disease, was not subjected to the same treatment. People could not be made to understand that this was not the fault of the Board of Health. It might be asked, why did we not prosecute all derelict doctors for violating the law? It can be readily understood that any doctor can entrench himself behind his diagnosis, and it would be impossible to convict him. All health records are necessarily at the mercy of the medical profession, and dependent on the skill, integrity and law-abiding spirit of practicing physicians An opportunity to verify the diagnosis is only afforded when the physician reports the case as suspicious.

Yellow fever was in Ocean Springs weeks before any suggestion or suspicion of its existence reached New Orleans. The cases were mild and not recognized by attending physicians. The same is true of earlier cases, which existed in New Orleans in July and August. These cases, if not recognized, and not reported to the Board of Health office, could not have been divined by intuition by the president and members of that board, and hence the board could not be blamed for the lack of early recognition. Censure for failing to recognize such cases is on a parity with other criticisms worked up and dished out to a clamoring and prejudiced public.

The same lawless spirit against constituted authority, which more than once has brought discredit on this community, seems to have pervaded the deliberations of the exchanges which conspired to depose this board. If succeeding boards hope to please these unreasoning, imperious masters, let them bury their sensibilities before assuming the guardianship of the public health of the State of Louisiana.

The flagging of premises, where infectious disease exists, is done in obedience to law; as officials, we have tried to carry out the law. The quarantining of premises is nothing new. It was practiced quite extensively in 1895, when we had small-pox in our city, but, as this disease was principally among negroes, and people in the lower walks of life, there was never any howl about the severities of the practice. Yellow fever, being no respector of person, and no prophylactic being known, has invaded houses of some of our clubmen, and members of the exchanges, and when their liberties are restricted, for the benefit of the town or State, they denounce the practice as a henious crime. Where is that boasted self-denial and devotion to duty of the New Orleans public? Does it exist in the laboring man only?

The practice of house quarantine was determined upon after consultation with the board of experts, the committee of the Orleans Parish Medical Society, the city officials, and voluntary committees of prominent citizens. It met with their entire approval. Has any one of these gentlemen come forward and offered to share the odium engendered by this practice? Has the press given voice to any conciliatory articles, and claimed its share in the responsibility for these measures? Have the mayor and city council intimated that these actions met their approval? And how about our friends among the exchanges, who labored with us night and day for the sake of shipping a few goods to the country? Their commerce was paralyzed, and they demanded a sacrifice.

We shall most willingly relinquish our thankless task, but in parting with our commercial friends, would remind them that charges are more easily made than proven, and that they have been charged with attempts at deception, shipping under false labels in violation of schedule agreed upon with quarantine authorities, and with sending circulars to the country denying the existence of yellow fever in New Orleans, when it was known throughout the Union.

With such charges against them, will their recent acts, demanding the resignation of the Board of Health, a board that enjoys the confidence of all neighboring authorities, for truth and sincerity at least, be calculated to restore public confidence and lessen the probability of this city being quarantined periodically for years to come?

